# Robust T-End Detection via T-End Signal Quality Index and Optimal Shrinkage

**DOI:** 10.3390/s20247052

**Published:** 2020-12-09

**Authors:** Pei-Chun Su, Elsayed Z. Soliman, Hau-Tieng Wu

**Affiliations:** 1 Department of Mathematics, Duke University, Durham, NC 27708, USA; peichun.su@duke.edu; 2 Department of Epidemiology and Prevention, Epidemiological Cardiology Research Center (EPICARE), Wake Forest School of Medicine, Winston-Salem, NC 27101, USA; esoliman@wakehealth.edu; 3 Section on Cardiovascular Medicine, Department of Internal Medicine, Wake Forest School of Medicine, Winston-Salem, NC 27101, USA; 4 Department of Statistical Science, Duke University, Durham, NC 27708, USA; 5 Mathematics Division, National Center for Theoretical Sciences, Taipei 106, Taiwan

**Keywords:** T-end annotation, signal quality index, tSQI, optimal shrinkage

## Abstract

An automatic accurate T-wave end (T-end) annotation for the electrocardiogram (ECG) has several important clinical applications. While there have been several algorithms proposed, their performance is usually deteriorated when the signal is noisy. Therefore, we need new techniques to support the noise robustness in T-end detection. We propose a new algorithm based on the signal quality index (SQI) for T-end, coined as tSQI, and the optimal shrinkage (OS). For segments with low tSQI, the OS is applied to enhance the signal-to-noise ratio (SNR). We validated the proposed method using eleven short-term ECG recordings from QT database available at Physionet, as well as four 14-day ECG recordings which were visually annotated at a central ECG core laboratory. We evaluated the correlation between the real-world signal quality for T-end and tSQI, and the robustness of proposed algorithm to various additive noises of different types and SNR’s. The performance of proposed algorithm on arrhythmic signals was also illustrated on MITDB arrhythmic database. The labeled signal quality is well captured by tSQI, and the proposed OS denoising help stabilize existing T-end detection algorithms under noisy situations by making the mean of detection errors decrease. Even when applied to ECGs with arrhythmia, the proposed algorithm still performed well if proper metric is applied. We proposed a new T-end annotation algorithm. The efficiency and accuracy of our algorithm makes it a good fit for clinical applications and large ECG databases. This study is limited by the small size of annotated datasets.

## 1. Introduction

The electrocardiogram (ECG) is a ubiquitous diagnostic tool for cardiovascular diseases. One important clinical application is information about the QT interval, a measure of ventricular repolarization. A prolonged heart rate corrected QT is associated with ventricular arrhythmia and sudden death [1], and also used to study adverse drug reactions [2].

The accuracy of QT measurement directly depends on the ability to accurately determine the Q onset and T offset. Compared with the Q onset, determination of the T-end is challenging. Nevertheless, various techniques have been proposed for automatic T-end detection. This includes threshold on the first derivative [3,4], threshold on an area connected by points around the T-wave [5,6,7], wavelet transform [8,9], mathematical model [10], support vector machine [11], artificial neural network (ANN) [12,13,14], hidden Markov model (HMM) [15,16], partially collapsed Gibbs sample and Bayesian [17], “wings” function [18], derivative curve [19], adaptive technique [20], TU complex analyses [21], correlation analysis [22], and k-nearest neighbor [23]. While those algorithms are widely applied, they are only validated on databases without severe noise contamination. It is thus unclear how robust they are to the inevitable noise and artifacts. The emerging wide use of long-term ECG recording that extends for days calls for robust automatic T-end annotation algorithms that handle marked prolonged noise such as myogenic noise, electrode contact noise, motion artifacts, and powerline interference [24,25].

In this report we propose a novel denoising tool to stabilize existing T-end annotation algorithms. The niche of the algorithm is twofold. First, we defined a signal quality index (SQI) that is suitable for the T-end annotation. This SQI describes the signal quality from a different perspective compared with other SQI aiming for R peak detection. Second, we applied a recently developed denoise technique called optimal shrinkage (OS) [26]. In our previous publication [27], we have demonstrated that a better and more adaptive template for each cardiac cycle is obtained through OS, which helps recover ECG morphology from noisy signals. However, we observed a morphology distortion after the application of OS on clean signals, where “clean” means the noise is dominated by the beat to beat variation. The noise level is thus overestimated and results in the distorted templates of cardiac cycle. Therefore, in this work, we incorporated the SQI thresholding step, such that the OS is only applied to signals with low signal quality. This part will be further addressed in the discussion section. Moreover, for those arrhythmic beats, we demonstrated that our algorithm still recovers their morphology when the proper metric between different cardiac cycles is selected.

## 2. Materials and Methods

### 2.1. Three Existing T-End Detection Algorithms

To keep things self-contained, we summarize three commonly applied T-end detection algorithms here. The algorithms are referred to by the last name of the first author, including Zhang [5], Carlos [6], and Martinez [8]. All these three algorithms require the locations of the QRS complex and T-wave before estimating T-end points. Both Zhang’s and Carlos’s algorithms are based on the idea that inside the designed searching window related to the detected R peaks, the maximum value of an area function is reached over the T-end point. Martinez’s algorithm uses dyadic wavelet transform to find the peaks and limit points of QRS cycles, P waves, and T waves. The T-end points are defined as the local maximum or minimum in the wavelet transform with dilation factor 2^4^ or 2^5^. These algorithms have been evaluated on the PhysioNet QT database [28] with low T-end detection errors. The performance of Martinez’s algorithm has also been validated on the dataset 3 of the CSE multilead measurement database (CSEDB) [29]. The MATLAB package of Zhang’s algorithm can be downloaded at http://www.irisa.fr/sosso/zhang/biomedical/ and Martinez’s algorithm can be downloaded at https://github.com/marianux/ecg-kit. We coded Carlos’s algorithm according to the original paper’s description. For T-wave locations, Zhang’s and Martinez’s algorithms have their built-in algorithms, so we simply adopted them. The T-wave locations estimated by Martinez were applied in Carlos. The other parameters of each algorithm were tuned in the same settings described in the original papers.

### 2.2. Signal Quality Index (SQI) for T-End Detection

Signal quality index (SQI) has been a critical quantity in the analysis of biomedical time series. It could be considered as an index summarizing the relationship between noise, artifact, and the signal of interest. In the ideal situation, if both the clean signal and the noise are clearly defined, the concept of signal-to-noise ratio (SNR) is a common approach to define SQI. However, usually it is challenging to get the clean signal and the noise or artifact from the given noisy signal, particularly when there is only one channel. Therefore, we need a different approach to determine the SQI.

**Figure 1:**
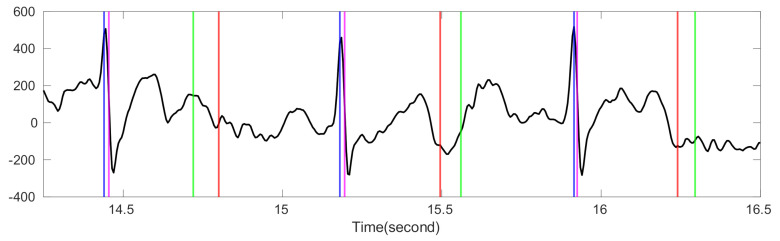
The comparison of bSQI and tSQI. The black line is the ECG signal from the Ultra-Long-Term ECG recordings Database with intermediate quality. The intersection of *x*-axis and blue (magenta) vertical lines are the time stamps of R peak locations estimated by Elgendi’s algorithm [34] (jqrs [30]). The intersection of *x*-axis and red (green) vertical lines are the time stamps of T-end locations estimated by Zhang’s algorithm (Martinez’s algorithm). The magenta vertical lines are shifted right by 10 milliseconds to avoid the overlap with blue lines. The value of bSQI is 0.99, and the value of tSQI is 0.66, which are calculated by matching points from two different algorithms as the same point if the time difference between them is smaller than 50 ms.

There have been various ideas in the past decades to define SQI for different biomedical signals [30,31,32,33]. The bSQI proposed by Li et al. [32] is a typical example. They applied two different R peak detection algorithms to the given ECG signal and obtained two sets of estimated R peak locations. If an ECG signal has a high signal quality, the estimated R-peak locations should be the same, or at least similar. This similarity is quantified as the SQI. This idea is simple but brilliant, and it can help us understand the quality of a given ECG signal. However, it only tells us the quality of the given ECG signal in the sense of R peak detection but not from all aspects. Specifically, it may not tell us how good the P wave is, or how clean the T wave is. See Figure 1 for an example. In this example we have a noisy ECG recording. Although R-peak locations estimated by two different R-peak detection algorithms, the Elgendi’s [34] and jqrs [30] algorithms, are similar, visually the T waves are not of high quality. Quantitatively, the T-end’s estimated by Zhang’s and Martinez’s algorithms are quite different. In summary, the bSQI only describes the signal quality from the aspect of R peak locations, and it cannot well manifest the signal quality for T-wave, and hence the T-end location.

Motivated by this observation, we propose a new SQI for the automatic T-end detection here. Similar to the idea behind the bSQI, we take two sequences of T-end locations detected by two T-end detection algorithms, and then determine the SQI in the following way. For each ECG segment, we assume that the R peak detection algorithm performs well and use the detected R peaks to estimate T-end points by two chosen algorithms. Denote *A* ∈N to be the number of detected R peaks and hence estimated T-end points. Given a grace period γ>0, we consider that two chosen algorithms match if the time difference between the two estimated T-ends is smaller than γ. Assuming we have *B* ∈N of T-end points that match, we denote a value tSQI $\in \left[0,1\right]$ as

$$\mathrm{tSQI}:\;=\;\frac{B}{2A-B}\;,$$

which is the desired SQI for the T-end detection. When tSQI = 1, it means that the two sequences of estimated T-end locations are perfectly matched, and hence the T-end quality is high; a lower tSQI stands for less matched estimations, and hence a lower T-end quality. In this work, we chose Zhang’s and Martinez’s algorithms to determine the tSQI in the following sections.

### 2.3. Proposed Algorithm 

We detail the propose automatic T-end annotation algorithm, step by step, here.

#### 2.3.1. Step 0: Preprocessing

Given an ECG recording, we resample it at the sampling rate 250 Hz. To remove the baseline wandering, a Butterworth lowpass filter of order 4 with the cut-off frequency $l_{off}$ Hz is applied. Since the source of data is unknown, we apply two notch filters with the notches centered at 50 and 60 Hz to reduce the powerline interference. The recording after the preprocessing is denoted as $z\in R^{N}$, where N∈N is the number of samples.

#### 2.3.2. Step 1: Estimate QRS Complex

There have been several works [30,34,35] discussing how to detect the location of QRS complexes. Since the T-end detection is our focus, here we simply use QRS locations estimated by Elgendi’s algorithm before applying any T-end detection algorithm. We denote the collected timestamps of estimated R-peak locations as

(1) $$R\;={\left\{R\left(i\right)\right\}}_{i=1}^{n}$$

where *R(i)* is the timestamp of the *i*-th detected R peak and n∈N is the total number of estimated R peaks.

#### 2.3.3. Step 2: Evaluate tSQI

For the *i*-th cardiac cycle, we evaluate its tSQI on a signal segment ${\left[z\left(R\left(i\right)-1250\right),\ldots ,z\left(R\left(i\right)+1250\right)\right]}^{⊤}$ with a grace period γ. Given a threshold number $q\in \left[0,1\right]$, if the tSQI ≥ q, the final estimated T-end of each cardiac cycle is determined by applying the desired T-end detection algorithm. Otherwise, the following steps are applied, and the final estimation would be made in Step 4.

#### 2.3.4. Step 3: Denoise Low Quality Cardiac Cycles by Optimal Shrinkage

- *Construct ECG templates.* On the *i*-th cardiac cycle determined by the *i*-th R peak, we find a window large enough, so that the whole P-QRS-T waveform is covered. For instance, denote w∈N to be the rounding number of the 95% quantile of R to R intervals in **R**, and denote the corresponding ECG segment over the cardiac cycle as
  (2) $$s_{i}:={\left[z\left(R\left(i\right)-L_{w}\right),\ldots ,z\left(R\left(i\right)+R_{w}\right)\right]}^{⊤}\in R^{p}$$
  where $L_{w}\in N,\;R_{w}\in N$, and $p:=L_{w}+\;R_{w}+1$. A library for the ECG template is built and denoted as
  (3) $$L:={\left\{s_{i}\right\}}_{i=1}^{n}$$
- *Remove nuisance variables by optimal shrinkage*. Define a metric $d(s_{a},\;s_{b}$) between the a-th and b-th template. For instance, we can use R-R interval difference in respect to the cycles or *l*_2_ norm $\left||s_{a}\;-s_{b}|\right|$*_l_*_2_ as the metric. For the *i*-th cardiac cycle $s_{i}$, from the library L we selecte ξ∈N neighbors that have the smallest difference. We then construct a data matrix S of size $p\;\times \left(\;\xi +1\right)$ consisting of the *i*-th cardiac cycle in the first column and all other neighbors in the remaining ξ columns. We assume that the S can be decomposed into two parts, S = *X + N*, where *X* is the clean data matrix containing QRS cycles and *N* is the matrix modeling noise. We also assume each entry of *N* has independent and identical noisy component with zero mean and the same variance and finite fourth moment, and *X* has a low rank. Under these assumptions, there is an elegant solution based on the random matrix theory proposed by Gavish and Donoho [26] to recover *X* from S**,** which is named as optimal shrinkage (OS). We apply OS on the data matrix S in the following manner:

Suppose $\frac{p}{\xi }\leq 1$ (if not, we simply take the transpose of S and apply the same approaches on $S^{⊤}$ as the following). Denote the SVD of the data matrix S as

(4) $$S=\sum_{i=1}^{r}\lambda_{i}u_{i}v_{i}^{\prime },$$

where r is the matrix rank, $u_{i}$ and $v_{i}^{\prime }$ are the *i*-th left and right singular vector corresponding to the singular value $\lambda_{i}$, and $\lambda_{1}\geq \lambda_{2}\geq \ldots \geq \lambda_{p}$. We normalize S with the noise level estimated by

(5) $$\sigma :=\sqrt{\frac{1}{n\cdot p}\sum_{i=1}^{n}\sum_{k=1}^{p}{\left(s_{i}\left(k\right)-\bar{s}\left(k\right)\right)}^{2}},$$

and

(6) $$\bar{s}\left(k\right)=\mathrm{median}\;\left\{s_{1}\left(k\right),\ldots ,s_{n}\left(k\right)\right\},$$

where k=1,…,p. Then, the denoised data matrix is estimated from S by OS and denoted as

(7) $${\tilde{S}}^{\eta^{*}}=\sigma \sum_{i=1}^{r}\eta^{*}\left(\frac{\lambda_{i}}{\sigma }\right)u_{i}v_{i}^{\prime },$$

where $\eta^{*}:\left[0,\infty \right)\to \left[0,\infty \right)$ is the optimal shrinker. In this work, we let $\eta^{*}$ be the optimal shrinker that minimize the asymptotic loss with respect to the operator norm, such that

(8) $$\eta^{*}\left(\lambda \right)=\frac{1}{\sqrt{2}}\sqrt{\lambda^{2}-\beta -1+\sqrt{{\left(\lambda^{2}-\beta -1\right)}^{2}-4\beta }}\;,$$

when $\lambda \geq 1+\sqrt{\beta }$ and $\eta^{*}\left(\lambda \right)=0$ otherwise. The first column of ${\tilde{S}}^{\eta^{*}}$ is the estimated QRS complex of $s_{i}$, denoted as ${\tilde{s}}_{i}$. Applying OS on every cardiac cycle in L, we acquire a denoised library and denote it as

(9) $$L:={\left\{{\tilde{s}}_{i}\right\}}_{i=1}^{n}.$$

#### 2.3.5. Step 4: Evaluate T-End Locations

As mentioned earlier, T-end detection algorithms of Zhang, Carlos, and Martinez are chosen for T offset delineation. Chosen T-end detection algorithm is applied in the library $\tilde{L,\;}$ and locations of T-end are estimated for each denoised cardiac cycles. Based on the R-peak locations **R** estimated in Equation (2), estimated T-end locations on the signal z are the final output of the proposed algorithm.

### 2.4. QT Database

The first database we considered is a common benchmark database, the PhysioNet QT database [28]. It includes ECG signals chosen from the MIT-BIH database [36] and the European ST-T database [37]. The database contains 105 subjects. Each subject has fifteen-minutes recordings of 2-channel ECG signals sampled at the 250 Hz, which include a broad variety of QRS and ST morphology. A subset of cardiac cycles in the database have been manually annotated with waveform boundaries, and only a small portion of signals was annotated for T-end. The manual annotations were made by two cardiologists using an interactive graphic display to view both signals simultaneously. The first cardiologist (Cardio 1) made annotations on all 105 subjects, and the second cardiologist (Cardio 2) made annotations on only 11 subjects. Following the same approach in Zhang’s work [5], we separated the QT database into three sets for analysis according to which cardiologists made the annotations. The first set (Set 1) contains all 105 subjects, in which we considered the 3542 annotations made by Cardio 1 as the true T-end points. The second set (Set 2) is from the 11 subjects annotated by both Cardio 1 and Cardio 2, in which we considered the 487 T-end points annotated by Cardio 1 as the ground truth. The third set (Set 3) also includes the same 11 subjects as Set 2, but the ground truth is the 402 annotations made by Cardio 2.

To evaluate our proposed algorithm under various noisy environment, we considered two additive noise models, the random Gaussian noise and the ARMA(1,1) noise [38], and contaminated the QT database by these noises. Assume the original signal is $z\in R^{N}$, where N∈N is the number of samples. The Gaussian noise was generated by N random points following the Gaussian distribution with mean 0 and variance 1. The ARMA (1,1) noise was generated by the ARMA (1,1) process:

$$X_{t}-\phi X_{t-1\;}=c+Z_{t}+\theta Z_{t-1},$$

where t=1,…, N is the sampling point, $X_{t}$ is the ARMA (1,1) process sampled at point *t,* $Z_{t}$ is a random variable following Student t-4 distribution sampled at point t, and c, ϕ and θ are selected constant. We set c = 0.5, ϕ = 0.5 and θ = −0.5 for following evaluation. The desired ARMA (1,1) noise at point *t* with variance 1 was then acquired by

$$X_{t}/SD(\{X_{t}{\}}_{t=1}^{N}),$$

where SD({$X_{t}{\}}_{t=1}^{N}$) is the standard deviation of the sequence {$X_{t}{\}}_{t=1}^{N}.$ Define a constant S

$$S:=\sqrt{P_{z}}/10^{\mathrm{SNR}/20},$$

where SNR is the desired signal to noise ratio and $P_{z}$ is the power of original signal. We then multiplied both Gaussian and ARMA (1,1) noise by S and added them to z to create the noisy signal with desired SNR. We evaluated SNR of 10 dB and 5 dB in the following evaluation.

### 2.5. 14-Day ECG Recordings Database

Our database of single-lead, ultra-long-term ECG recordings comprised of four recordings; each recording is approximately two weeks (14 days) in length. The data was recorded using the ZIO^®^ Patch cardiac monitor (iRhythm Technologies, Inc., San Francisco, CA, USA) at a sampling rate of 200 Hz. The underlying information of the subjects was unknown to us. Across the four 14-day recordings, we randomly selected 51, 11, 24, 80, and 32 segments of 10 s for manual review and annotation at the Epidemiological Cardiology Research Center (EPICARE Center, Wake Forest School of Medicine, Winston Salem, NC, USA). The ECG core laboratory was provided with the automated T annotation generated by our algorithm, and the results were reviewed by the ECG core readers for accuracy and editing. The quality of each ECG segment was also documented as part of the annotation process. In total, 173 segments were labeled good, 16 segments were labeled intermediate, and 9 segments were labeled bad.

### 2.6. Evaluation

We evaluated our proposed T-end annotation algorithm, which is an enhancement of Zhang, Carlos, or Martinez, on the above-mentioned databases. For each ECG recording, we cropped a segment for analysis, which started from 1 min before the first T-end annotations and ended at 1 min after the last T-end annotation. The R-R interval difference between cardiac cycles was applied as the metric $d(s_{a},\;s_{b}$). Since our focus was automatically annotating T-end, the R peaks were assumed to be known. The T-end detection error is the time difference between the automatically detected T-end points and the provided annotations. In the QT database, since each subject has two recordings, the estimated T-end points were chosen from the best result that minimized the detection error among the two computed T-end positions. This approach is the same as that used by Zhang and Carlos. The justification of this approach is that the cardiologists made their annotations by evaluating both ECG records. For the Ultra-Long-Term ECG recordings database, since there is only one channel for each recording, we simply calculated the estimated T-end locations and provided annotations.

In our previous work, the effects of noise robustness and morphology recovery by optimal shrinkage and other template estimation algorithms had been compared for the purpose of extracting fetal ECG from the trans-abdominal maternal ECG [27] over the Physionet CinC Challenge 2013 database [39]. These methods included independent component analysis (ICA), principal component analysis (PCA), least mean square (LMS), recursive least square (RLS), echo state neural network (ESN), and extended Kalman filter (EKF). To further demonstrate the strength of optimal shrinkage, in this work we compared with another ECG denoising method named wavelet shrinkage [40,41]. The idea is similar to the optimal shrinkage, where the empirical wavelet coefficients are shrunk, and the signals are recovered by the inverse wavelet transform. The main difference is that in OS, the basis is adaptive to the data, while in the wavelet shrinkage, the basis is predetermined. We compared these two algorithms on the QT database with the additive Gaussian or ARMA (1,1) noise. The wavelet shrinkage is applied after *Step 0: preprocessing*, where wavelet shrinkage is applied on the denoised signal $z\in R^{N}$ following the setup detailed [42]. Then, *Step 1* and *Step 4* are applied for R peaks and T offset delineation.

To evaluate the performance of each algorithm, the mean (ME) for the absolute values of detection errors of each recording were calculated. Then, the median and MAD and 2.5–97.5% quantile interval of ME over all recordings were computed and reported. To evaluate if the proposed OS approach improved the existing algorithm, we applied the Wilcoxon signed rank test with the statistical significance level set to 0.05.

## 3. Results

The parameters of proposed algorithm were set as follows. We set the grace period γ= 50 ms for the tSQI evaluation. Moreover, we set the cutoff frequency for low pass filter as $l_{off}$ =0.5 Hz, window length $L_{w}=R_{w}=\lceil \frac{1}{2}\rceil$, where ⌈x⌉ is the smallest integer larger than x, and number of neighbors ξ = 19. The evaluation was performed on MATLAB 2019b, Microsoft 10 system, Intel i7-6700HQ CPU@2.60GHz 4 cores, and 16GB RAM. The code can be downloaded at: https://github.com/MagineZ/Tend-Project.

### 3.1. tSQI and Quality in the 14-Day ECG Database

The comparison of the signal quality provided in the 14-day ECG database and the proposed tSQI in the ultra-long ECG recording database is reported in Table 1. We observe that the ECG signals labeled as good (or bad) quality have higher (or lower) tSQI’s. If we view label good as 1, label intermediate as 3, and label bad as 5, the correlation coefficient between the tSQI and labeled quality is −0.71.

### 3.2. Noise Suppression

**Figure 2:**
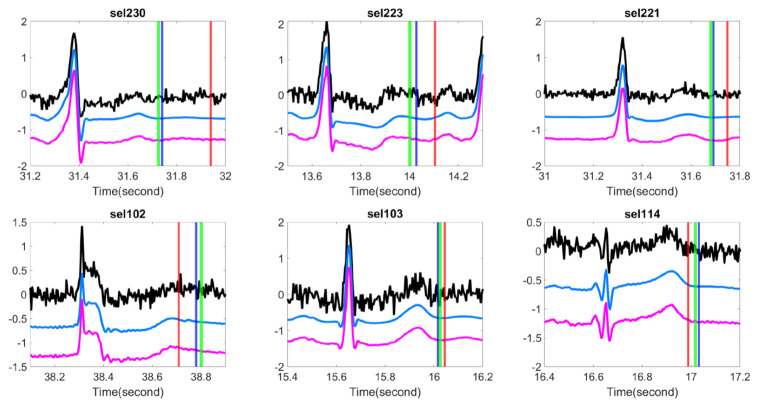
Comparison of different T-end detection algorithms with or without optimal shrinkage when the signal is contaminated by the additive Gaussian noise. In each subplot, the black line is the ECG signal from the first channel of a subject in the QT database with additive noise, the light blue line is the denoised ECG signal by the optimal shrinkage, and the magenta line is the original ECG signal without adding any noise. The red (green) vertical lines indicate the estimated T-end by the chosen detection algorithm on noisy signals without (with) OS applied. The deep blue vertical line indicates the annotated T-end by Cardio 1. The plots in the first (second) row are the results when the SNR is 10 dB (5 dB). The chosen T-end algorithm in the first, second and third column is Zhang’s, Carlos’s, and Martinez’s algorithms respectively. The case index of each plot is in the title.

**Figure 3:**
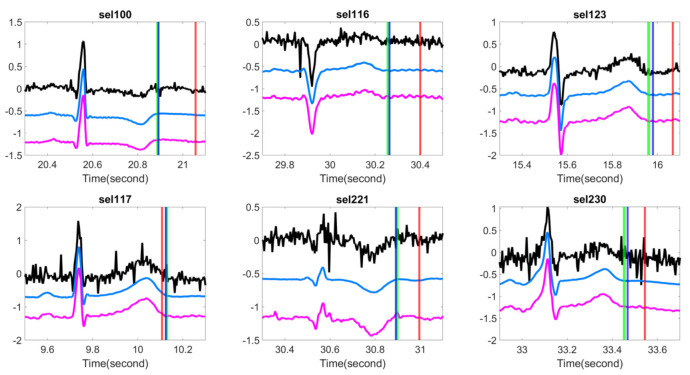
Comparison of different T-end detection algorithms with or without optimal shrinkage when the signal is contaminated by the additive ARMA (1,1) noise. In each subplot, the black line is the ECG signal from the first channel of a subject in the QT database with additive noise, the light blue line is the denoised ECG signal by the optimal shrinkage, and the magenta line is the original ECG signal without adding any noise. The red (green) vertical lines indicate the estimated T-end by the chosen detection algorithm on noisy signals without (with) OS applied. The deep blue vertical line indicates the annotated T-end by Cardio 1. The plots in the first (second) row are the results when the SNR is 10 dB (5 dB). The chosen T-end algorithm in the first, second and third column is Zhang’s, Carlos’s, and Martinez’s algorithms respectively. The case index of each plot is in the title.

We examined if the proposed OS denoising could help stabilize existing T-end detection algorithms under various noise types and noise levels. The results for different SNR levels are reported in Table 2 for the Gaussian noise and ARMA (1,1) noise respectively. It is clear that the proposed OS algorithm helps improve the traditional algorithms and outperforms wavelet shrinkage. Wilcoxon signed rank test over the 20 rounds’ rejects the null hypothesis with statistical significance. To appreciate how the OS recovers the ECG signal, some examples are shown in Figure 2 and Figure 3. We see that the deviations of estimated T-end from the annotation is obvious on the signals without OS applied, and the OS helps recover the ECG morphology and thus reduces the automatic annotation errors. This suggests the potential of the proposed denoise algorithm to help stabilize existing algorithms.

### 3.3. Evaluation in the QT and 14-Day ECG Databases

**Figure 4:**
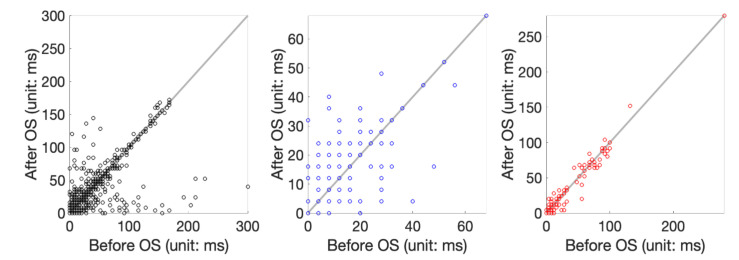
Scatterplot of T-end prediction error before and after applying the OS. From left to right are the scatterplot of applying Zhang’s algorithm to the set 1, set 2, and set 3 of the QT database.

We applied the proposed tSQI and OS denoising to the QT and 14-day ECG recording databases. We considered tSQI thresholds *q* = 0.9 when applying the proposed algorithm; that is, the OS was applied to those cardiac cycles with the tSQI less than 0.9. Note that *q* = 0 means we do not apply the OS. The results are shown in Table 3 and Table 4. First, note that in Zhang’s and Carlos’s algorithms, the ME and SD were defined without taking the absolute value. The error is thus underestimated since the detection error of the estimated T-end has positive or negative values. Therefore, smaller MEs were reported by Zhang and Carlos in comparison with our results when *q* = 0, even if we applied their announced codes on the same database. We see that, overall, the MAD and 97.5% quantile are improved, while there is no statistical significance. To have a better feeling about how the algorithm performs, the scatterplots of T-end detection errors of the QT database are shown in Figure 4.

## 4. Discussion

In this report, we showed that the labeled quality in the 14-day ECG database provided by the ECG core lab is well captured by the proposed tSQI. We also showed how the noise deteriorates the signal quality and thus induces high detection errors in both databases when the commonly applied T-end detection algorithms are applied. The proposed OS denoise was able to recover the ECG morphology when the signal was contaminated by noise, and hence stabilized the T-end detection algorithm. In the arrhythmic cases, the T-wave morphologies could be visually better recovered by selecting proper metric between cardiac cycles.

**Figure 5:**
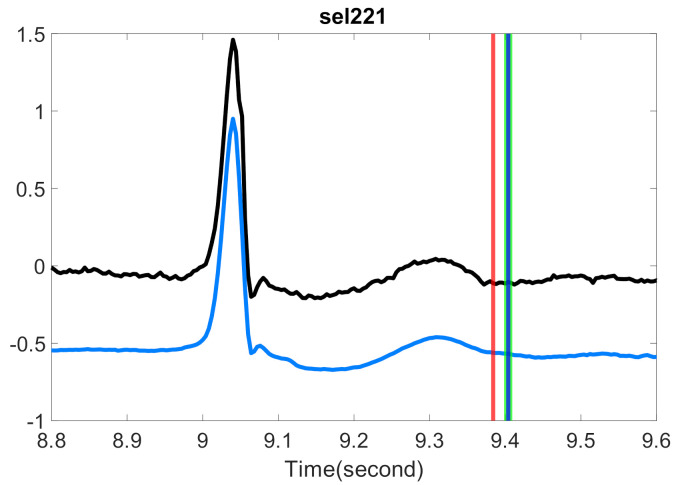
Comparison of the estimated T-end with or without optimal shrinkage on P-QRS-T cycles with high signal quality in the QT database. The black line is the preprocessed ECG signals from the first channel of one subject, and the light blue line is the same signals with the optimal shrinkage applied, where the whole signal is shifted down by 0.5 to enhance visualization. The red vertical line indicates the T-end point estimated by the chosen algorithm on the original signal. The green vertical line indicates the T-end point by the chosen algorithm on the denoised signal. The deep blue vertical line indicates the time point of T-end annotations provided by Cardio 1. Zhang’s algorithm is applied for the T-end detection. The case index is in the title.

**Figure 6:**
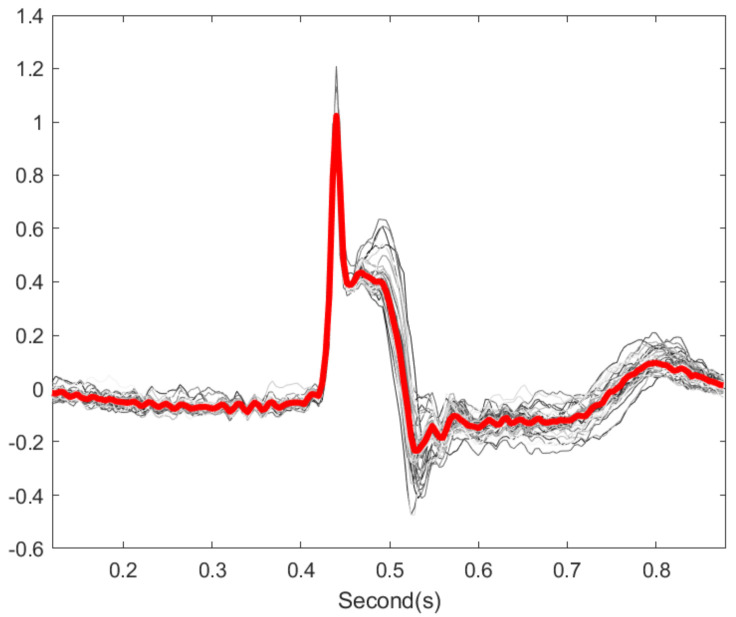
Illustration of cardiac cycles variation. The gray lines are 20 cardiac cycles from subject id sel30 in QT database. The red line is the median over the 20 cycles.

The spike model underlying the proposed algorithm deserves discussion. As shown in Figure 5, there is T-wave distortion after the OS, and hence the T-end location shifts. In other words, when the ECG signal is clean, a morphology distortion after the OS is applied. This observation suggests that the cardiac cycles cannot be fully captured by the spike model used in Gavish’s and Donoho’s work [26] for the OS, and hence the morphology is distorted when the noise is dominated by the beat to beat variation. Recall that the PQRST morphology is not fixed from cycle to cycle, and the variation is caused by various physiological dynamics such as the respiratory and heart rate [2]. See Figure 6 for an example, where 20 cycles of clean PQRST waves from the same subject were superimposed. It is also clear that the PQRST variation from cycle to cycle is small compared with the dominant PQRST pattern. Therefore, the data matrix for a set of clean cardiac cycles can be modeled by $E=\sum_{i=1}^{p}\mu_{i}e_{i}a_{i}^{\prime }\in R^{p\times n}$, where $\mu_{1}\gg \mu_{2}\geq \mu_{3}\geq \cdots$, $e_{1}\in R^{p}$ is the dominant PQRST pattern, $a_{1}\in R^{n}$ describes the magnitude the dominant PQRST pattern of each cycle, $e_{2}\ldots e_{p}\in R^{p}$ describe the variation of the PQRST pattern, and $a_{2}\ldots a_{p}\in R^{n}$ describe the magnitude of each variation of the PQRST pattern. Clearly, E can be well approximated by a spike model $\tilde{E}=\mu_{1}e_{1}a_{1}^{\prime }\in R^{p\times n}$. While this seems to be a nice approximation, it does generate trouble when we apply the OS. Specifically, when the signal is clean, Equation (5) for noise level estimation will consider the physiological variation $\sum_{i=2}^{p}\mu_{i}e_{i}a_{i}^{\prime }$ between each PQRST cycle as a source of noise. Thus, the noise level of the clean signal is overestimated. As a result, the OS might eliminate $\sum_{i=2}^{p}\mu_{i}e_{i}a_{i}^{\prime }$ in order to recover the “clean matrix” $\mu_{1}e_{1}a_{1}^{\prime }$, which leads to a distortion of morphology. The critical observation is that the variation of T wave morphology from cycle to cycle is quantified by the variation term $\sum_{i=2}^{p}\mu_{i}e_{i}a_{i}^{\prime }$. However, due to the nonlinear relationship among T waves from cycle to cycle, it is not clear how to directly use the T wave information in $\sum_{i=2}^{p}\mu_{i}e_{i}a_{i}^{\prime }$. When the signal is heavily contaminated by noise, $\bar{E}=\sum_{i=1}^{p}\mu_{i}e_{i}a_{i}^{\prime }+\xi$, where ξ is the noise matrix, the small variation term $\mu_{2}e_{2}a_{2}^{\prime },\ldots ,\mu_{p}e_{p}a_{p}^{\prime }$ will be dominated by the noise, particularly those with small singular values. Hence, there is benefit from applying the OS because it will balance between recovering the morphology and reducing the noise. This explains why we only apply OS to those cycles with low tSQI. We mention that in general, the dynamics of T wave morphology can be modeled by a low dimension manifold, which is referred to by the wave-shape manifold [43]. An exploration of this model to further improve the denoising performance, or even noise estimation shown in Equation (5), is out of the scope of this paper.

**Figure 7:**
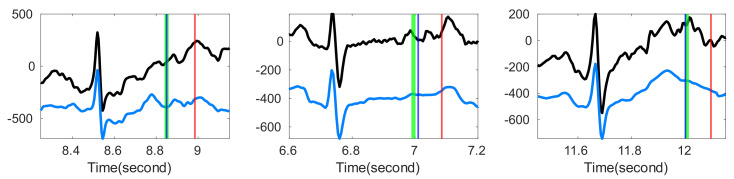
Comparison of the estimated T-end points with or without optimal shrinkage over Ultra-Long-Term ECG recordings Database with intermediate quality. In each subplot, the black line is the original ECG signal, and the blue line is the denoised ECG signal estimated by the optimal shrinkage. The red (green) vertical lines indicate the estimated T-end by the chosen detection algorithm on noisy signals without (with) OS applied. The deep blue vertical line indicates the annotated T-end provided by EPICARE Center. The chosen T-end algorithm in the first, second and third column is Zhang’s, Carlos’s, and Martinez’s algorithm respectively.

In the QT and ultra-long-term ECG recordings databases, while there was improvement of the T-end algorithm after applying OS with a properly chosen tSQI threshold, this improvement did not reach statistical significance. See Table 3. There are two possible explanations. First, the motion or other artifacts are inevitable. Usually, artifacts have structures that are unknown to us, unless we have other ECG channels to confirm. Due to these artifacts, visual manual annotation of T-end might be challenging. Consider the following scenario. Suppose the visual manual annotation of T-end was deviated by the existence of noise or artifacts. On the other hand, the denoising tool recovered the T wave, and hence the automatic T-end annotation algorithm provided the correct T-end annotation. In this case, the “correctly detected T-end” will be viewed as a bad annotation compared with visual manual annotation of T wave. To visualize this effect, we show some results in Figure 7. When the signal quality is labeled as average in the ultra-long-term ECG recording database, it is challenging even for experts to annotate the T-end due to noise and artifacts.

Second, the OS performance might be masked by the experts’ annotation process. This can be observed clearly in the ultra-long-term ECG recording database. Due to the labor-intensive annotation process, the data analysis team provided the automatic annotated labels by applying Zhang’s algorithm, and the experts only provided a corrected T-end when they disagreed with the automatic label. This “semi-automatic” process might bias the experts’ T-end annotation. We can see this potential bias in Table 4. Before applying the OS, the Zhang’s algorithm outperforms other algorithms with or without OS. Therefore, we may conclude that it is possible that the non-statistical significance comes partially from the label uncertainty issue. Combining these facts and its performance when marked noise of different kinds exists, we may argue that the proposed stabilization algorithm by combining tSQI and OS is potential for automatic T-end annotation purpose in practice. We may also discuss a relevant but different topic. The labeling procedure for the ultra-long-term ECG recording database in this study can be viewed as an artificial intelligence aided approach to speed up the standard annotation procedure. By and large, the common labeling procedure is composed of two steps. First, one group of experts provide an initial manual annotation of ECG waveform. Then, another group of experts doubly confirm the provided annotation and edit extreme values as a quality control. In this study, we replace the first step by the existing best automatic annotation system and control the overall quality by a human expert intervention. How to improve this semi-automatic labeling procedure to avoid the above-mentioned issues is of its own interest, and it will be explored in our future work.

**Figure 8:**
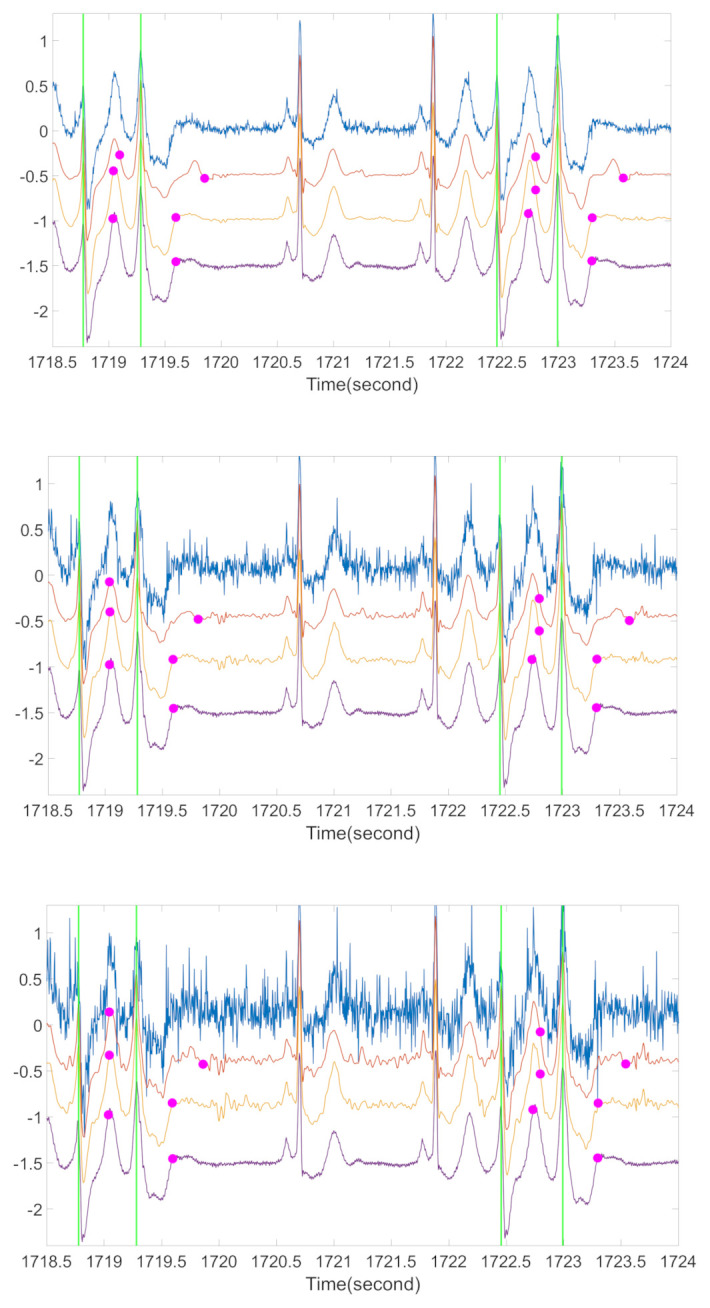
A preliminary comparison of ECG morphologies with different metrics for arrhythmic ECG. For each subplot, the blue line is the ECG contaminated by ARMA (1,1) noise, the orange line is the ECG after OS with RRI as the metric, the yellow line is the ECG after OS with *l*_2_ norm as the metric, the purple line is the original ECG, green vertical lines are the locations of R peaks in each PVC occurrence, and the magenta spots are the corresponding estimations of T-end locations by Zhang’s algorithm. The signal is from the first channel of case 106 in MITDB. From top to bottom subplot, the noise level is 20 dB, 10 dB, and 5 dB respectively.

Another topic deserves a discussion is arrhythmic ECGs. It is well-known that occasional arrhythmias, like premature ventricular contraction (PVC), can cause sudden T wave changes in normal rhythms. Although we cannot find a publicly available arrhythmia database with T-end annotation, we provide some preliminary visual evaluation of the OS algorithm on the ECG signals with arrhythmia. We consider the Physionet MITDB [44] that contains 48 half hour excerpts of two-channel ambulatory ECG recordings. Each recording has 2 channels with the sampling frequency 360 Hz and the 11-bit resolution over a 10-mV range. The R peak annotations are provided. To see the performance of our proposed algorithm on arrhythmic ECGs, we applied OS on MITDB with Zhang’s method for T-end annotation. Both the R-R interval difference and the *l*_2_-norm of difference between cardiac cycles were applied as the metric $d(s_{a},\;s_{b}$). See Figure 8 for a typical result. We see that the distortion of T-wave morphology is obvious when the RRI metric is applied. The distortion is less when the l_2_ norm is used as the metric to compare two cardiac cycles. For example, for the cycles around 1719.2 s and 1723 s, the T wave is distorted when the RRI metric is applied, and these distortions disappear when the l_2_ norm is considered and the ECG signal is better recovered. While we do not have experts’ label for T end, visually when the *l*_2_ norm is applied, the detected T ends are closer to those detected from the original ECG signal, while the detected T-ends might not be correct. This preliminary result is encouraging, but an extensive study is needed to evaluate its performance. While developing a more accurate T-end detection algorithm is not the focus of this paper, we mention that developing such an algorithm that is suitable for arrhythmic ECG is another critical topic for exploration.

There are several limitations in this study. First, the labeled database is small. Because manual T-end labeling for ultra-long ECG recording to validate the algorithm is a labor intense process, even with the help of the initial labels, the sample size was small. Second, we need to develop a new denoising algorithm that can handle structured artifacts. The main challenge is how to model artifacts when there is no extra information. A basis pursuit approach might be suitable, and we will report our theoretical research result in our future work. Third, in this article we focus on the tSQI and ECG denoising. To further improve the T-end annotation accuracy, we may need to further improve existing T-end annotation algorithms, probably by incorporating some machine learning algorithms.

In conclusion, our proposed T-end annotation algorithm has the efficiency and accuracy that make it a good fit for clinical applications and large ECG databases.

## Figures and Tables

**Table 1:** The relationship between the tSQI and quality.

Labeled Quality	Good	Average	Bad
tSQT	0.98 ± 0.06	0.84 ± 0.12	0.68 ± 0.08

Note: If we view label good as 1, label intermediate as 3, and label bad as 5, the correlation coefficient between the tSQ and labeled quality is −0.71.

**Table 2:** Evaluation of algorithms on the QT database under contamination of Gaussian/ARMA (1,1) noise.

	**SNR**	**Zhang**					
		**Gaussian**			**ARMA (1,1)**		
		***q* = 0**	**WS**	***q* = 0.9 (OS)**	***q* = 0**	**WS**	***q* = 0.9 (OS)**
Set 1	10 dB	22.32 ± 0.26	20.15 ± 0.24	17.43 ± 0.19 **	22.39 ± 0.22	20.15 ± 0.22	17.53 ± 0.17 **
		[21.47, 22.99]	[19.58, 20.75]	[16.97, 17.76]	[21.47, 22.60]	[19.64, 20.74]	[17.16, 18.01]
	5 dB	29.22 ± 0.51	23.84 ± 0.33	18.35 ± 0.44 **	28.55 ± 0.38	24.13 ± 0.35 **	18.57 ± 0.49 **
		[28.12, 30.]	[22.82, 24.25]	[17.62, 19.30]	[27.97, 29.57]	[23.62, 25.02]	[17.71, 19.65]
Set 2	10 dB	17.91 ± 0.67	16.81 ± 0.51	15.35 ± 0.47 **	17.95 ± 0.75	16.65 ± 0.56 **	15.20 ± 0.46 **
		[15.95, 20.04]	[15.65, 17.85]	[14.07, 16.16]	[15.68, 19.24]	[15.87, 18.78]	[14.41, 16.92]
	5 dB	23.96 ± 0.61	19.68 ± 0.62	16.08 ± 0.43 **	23.23 ± 1.01	19.76 ± 0.88	15.66 ± 0.70 **
		[22.85, 25.55]	[18.49, 21.16]	[15.04, 17.18]	[21.48, 26.42]	[18.21, 21.78]	[15.09, 18.89]
Set 3	10 dB	21.85 ± 1.63	19.81 ± 1.22	20.33 ± 0.50 *	22.36 ± 1.06	20.22 ± 0.60 *	20.07 ± 0.57 *
		[20.06, 27.38]	[18.55, 23.38]	[18.74, 21.09]	[19.97, 24.42]	[17.87, 22.54]	[18.79, 21.91]
	5 dB	26.85 ± 1.16	21.40 ± 1.69	21.43 ± 0.62 **	26.87 ± 1.33	22.77 ± 1.30	20.82 ± 0.67 **
		[24.99, 30.09]	[19.13, 27.01]	[19.52, 23.02]	[23.33, 31.10]	[19.36, 25.74]	[19.96, 24.22]
		**Carlos**					
		**Gaussian**			**ARMA (1,1)**		
		***q* = 0**	**WS**	***q* = 0.9 (OS)**	***q* = 0**	**WS**	***q* = 0.9 (OS)**
Set 1	10 dB	25.12 ± 0.34	24.70 ± 0.34	22.78 ± 0.33 **	25.82 ± 0.28	25.36 ± 0.26	22.98 ± 0.28 **
		[24.41, 25.93]	[24.07, 25.42]	[22.18, 23.58]	[25.13, 26.51]	[25.03, 26.00]	[22.35, 23.86]
	5 dB	32.27 ± 0.41	29.12 ± 0.30	23.19 ± 0.45 **	32.53 ± 0.57	29.75 ± 0.45	23.39 ± 0.27 **
		[31.28, 32.86]	[28.31, 29.63]	[21.96, 24.44]	[30.83, 34.14]	[28.69, 31.01]	[22.53, 23.90]
Set 2	10 dB	21.20 ± 0.71	20.10 ± 0.56	19.49 ± 0.79 **	22.01 ± 0.93	20.59 ± 0.67	19.56 ± 0.63 *
		[19.01, 22.22]	[17.56, 20.61]	[16.81, 21.47]	[19.18, 23.53]	[19.09, 22.97]	[18.67, 21.45]
	5 dB	25.62 ± 0.75	23.14 ± 0.75	19.99 ± 1.10 **	26.61 ± 1.10	25.46 ± 0.70	20.29 ± 0.87 **
		[23.45, 26.81]	[20.43, 24.67]	[16.41, 22.16]	[24.91, 29.69]	[23.20, 26.88]	[18.32, 22.28]
Set 3	10 dB	19.96 ± 0.98	17.15 ± 1.28	14.67 ± 0.54 **	20.33 ± 1.14	18.32 ± 1.25	14.62 ± 0.78 **
		[17.89, 24.02]	[14.37, 19.65]	[13.22, 15.99]	[18.04, 22.90]	[15.35, 20.59]	[13.76, 17.12]
	5 dB	26.12 ± 1.24	21.15 ± 1.30	16.03 ± 0.83 **	27.21 ± 1.74	23.72 ± 1.06	15.67 ± 0.92 **
		[23.56, 31.77]	[18.50, 24.36]	[14.07, 17.69]	[23.51, 31.32]	[20.58, 26.85]	[13.51, 18.45]
		**Martinez**					
		**Gaussian**			**ARMA (1,1)**		
		***q* = 0**	**WS**	***q* = 0.9 (OS)**	***q* = 0**	**WS**	***q* = 0.9 (OS)**
Set 1	10 dB	26.64 ± 0.29	25.74 ± 0.26	22.86 ± 0.25 **	26.35 ± 0.22	25.47 ± 0.25	22.91 ± 0.26 **
		[26.16, 27.29]	[25.01, 26.25]	[22.22, 23.54]	[25.77, 26.80]	[24,75 25.85]	[22.13, 23.15]
	5 dB	33.87 ± 0.29	30.38 ± 0.30	23.39 ± 0.34 **	33.51 ± 0.24	30.17 ± 0.30	23.43 ± 0.48 **
		[33.04, 34.38]	[29.40, 31.35]	[22.43, 24.41]	[32.42, 33.84]	[29.40, 30.90]	[22.85, 24.86]
Set 2	10 dB	19.49 ± 0.73	17.41 ± 0.68	17.12 ± 0.79 **	19.24 ± 0.69	17.30 ± 0.43	17.02 ± 0.55 **
		[17.88, 21.35]	[15.83, 18.84]	[14.91, 18.69]	[17.38, 20.61]	[16.18, 18.14]	[15.91, 18.76]
	5 dB	25.67 ± 0.83	21.33 ± 0.84	17.52 ± 0.72 **	25.38 ± 0.79	21.03 ± 0.96	16.93 ± 0.74 **
		[23.19, 27.49]	[17.89, 22.92]	[16.29, 19.33]	[23.75, 27.94]	[18.78, 23.25]	[14.87, 18.45]
Set 3	10 dB	20.20 ± 0.97	17.38 ± 0.88	16.87 ± 0.79 **	20.12 ± 1.03	18.12 ± 1.18	17.55 ± 0.81 *
		[18.30, 23.09]	[15.71, 21.57]	[15.40, 18.52]	[18.27, 21.79]	[15.36, 21.93]	[14.58, 18.80]
	5 dB	27.12 ± 1.39	22.30 ± 1.61	18.77 ± 0.77 **	26.62 ± 1.62	23.02 ± 1.71	18.57 ± 0.79 **
		[23.92, 30.58]	[17.96, 27.41]	[16.51, 20.09]	[24.09, 32.05]	[19.46, 27.78]	[16.69, 19.58]

Note: Column one stands for which set is used for error computation. Column 2 is the signal to noise ratio. Column 3-5 (Gaussian noise) and 4-6 (ARMA(1,1) noise) are the results of T-wave end detection error using algorithms of Zhang, Carlos, and Martinez respectively, where q stands for the tSQI threshold for deciding when to apply the optimal shrinkage, WS stands for wavelet shrinkage, and OS stands for optimal shrinkage. The results are shown by taking median ± MAD of ME over 20 rounds’ random noise addition. The unit is millisecond. The 2.5% and 97.5% quantile interval of ME are also listed beneath median ± MAD. * (**) stands for p-value < 0.05 (0.01).

**Table 3:** Evaluation of algorithms on the QT database.

		Zhang		Carlos		Martinez	
	# of Beats	Before OS	After OS	Before OS	After OS	Before OS	After OS
Set 1	1417	12.00 ± 20.83	12.00 ± 18.96	12.00 ± 35.82	12.00 ± 32.84	12.00 ± 40.59	12.00 ± 37.88
		[0, 128.00]	[0, 116.00]	[0, 212.30]	[0, 224.00]	[0, 304.60]	[0, 332.00]
Set 2	169	12.00 ± 9.43	16.00 ± 9.17	12.00 ± 20.41	12.00 ± 19.94	12.00 ± 20.33	12.00 ± 18.47
		[0, 44.80]	[0, 44.00]	[0, 108.00]	[0, 101.60]	[0, 92.00]	[0, 84.80]
Set 3	132	16.00 ± 27.49	12.00 ± 27.37	8.00 ±14.22	8.00 ± 13.35	16.00 ± 22.60	12.00 ± 21.87
		[0, 100.00]	[0, 100.80]	[0, 72.00]	[0, 49.6]	[0, 79.20]	[0, 81.60]

Note: Column one indicates which set is analyzed. Column 2 is the number of heart beats. Column 3, 4, and 5 are the results of T-wave end detection error using algorithms of Zhang, Carlos, and Martinez respectively. The median ± mean absolute deviation (MAD) of detection errors are evaluated. The unit is millisecond. Note that since the sampling rate is 250 Hz, the median error is the multiple of 4 ms.

**Table 4:** Evaluation of algorithms on the Ultra-Long-Term ECG recordings database.

Labeled Quality	# of Beats	Zhang		Carlos		Martinez	
		Before OS	After OS	Before OS	After OS	Before OS	After OS
Good	538	0.00 ± 17.05	5.00 ± 13.55	5.00 ± 13.65	5.00 ± 12.95	10.00 ± 14.09	10.00 ± 13.68
		[0, 125.00]	[0, 115.25]	[0, 105.00]	[0, 95.50]	[0, 120.25]	[0, 115.50]
Averaged	148	0.00 ± 33.99	10.00 ± 29.94	10.00 ± 33.88	15.00 ± 38.23	10.00 ± 33.73	15.00 ± 34.10
		[0, 168.00]	[0, 159.00]	[0, 145.00]	[0, 200.00]	[0, 157.00]	[0.00, 169.00]
Bad	127	0.00 ± 45.04	30.00 ± 47.90	25.00 ± 50.46	35.00 ± 50.38	35.00 ± 54.88	50.00 ± 50.07
		[0, 208.25]	[0, 208.25]	[0, 236.63]	[0, 230.00]	[0, 216.63]	[0, 225.00]

Note: Column one indicates which set is analyzed. Column 2 is the number of heart beats. Column 3, 4, and 5 are the results of T-wave end detection error using algorithms of Zhang, Carlos, and Martinez respectively. The median ± mean absolute deviation (MAD) of detection errors are evaluated. The unit is millisecond. Note that since the sampling rate is 200 Hz, the median error is the multiple of 5 ms.
